# Trust has a price?! Unraveling the dynamics between trust in the media and the willingness to pay in the post-pandemic scenario

**DOI:** 10.1177/14648849241311101

**Published:** 2024-12-23

**Authors:** Denise Voci, Matthias Karmasin, Sonja Luef, Sandra Förster, Andy Kaltenbrunner

**Affiliations:** 191581 University of Klagenfurt, Klagenfurt, Austria; 31390 Austrian Academy of Sciences/ University of Klagenfurt, Vienna/Klagenfurt, Austria; Austrian Academy of Sciences,Vienna, Austria; 31390 Medienhaus Wien (MHW), Vienna, Austria

**Keywords:** Media trust, willingness to pay, payment intent, media expenditure, media brand trust, source credibility, message credibility

## Abstract

This study examines the link between media trust and consumers’ willingness to pay (WTP) for online news in the post-pandemic era. A 2023 survey of 1000 Austrian participants investigated how trust affects WTP and identified key predictors and moderators. Findings reveal a strong correlation between media trust and both WTP and media expenditure (ME), highlighting trust’s critical role in media organizations’ financial sustainability, as consumers favor credible news and trusted brands. Accordingly, media companies must develop a comprehensive strategy to (re)build trust, emphasizing the unique quality of their content and fostering trust in their brands to encourage paid over free content. By following this dual approach, media companies can adapt to the digital landscape, meet consumer expectations, and ensure economic and democratic sustainability.

## Introduction

The contemporary media industry faces a complex array of challenges, exacerbated by the disruptions brought about by the COVID-19 pandemic. The pandemic not only disrupted global economies but also accelerated the digital transformation of media consumption, creating a highly intricate post-pandemic media landscape. In this evolving environment, two critical issues persist, demanding particular attention: the decline in public trust in media and the ever-pressing need to (re)finance media products.

Trust in (news) media is not a new issue arising from recent populist attacks but has been studied for nearly a century (Fisher, 2016). The practice of “blaming the press” has a long history, with skepticism toward news media predating social media and becoming evident since the 1970s. This decline has profound implications, as media trust underpins democracy, fosters informed citizenship, and mitigates misinformation and disengagement (Lewis et al., 2008). Media shapes societal understanding and shared concerns (Coleman, 2012), and its erosion disrupts societal orientation, rational decision-making, and public acceptance of critical policies (Tsfati and Cohen, 2005).

Moreover, trust is essential for the media industry’s economic viability, as untrusted products struggle in the market, and an untrustworthy industry deters stakeholders (Schranz et al., 2018), worsening its pre-existing economic challenges. In fact, the media industry also faces complex challenges beyond trust issues, intensified by the Internet, the digital revolution, and platform disruptions to traditional revenue models reliant on advertising and sales (Schudson, 2022).

The COVID-19 pandemic has worsened both issues. While news consumption rose in the pandemic’s first year (Newman, 2021), it did not boost trust or financial stability. Media institutions remain the least trusted, ranking below corporations, NGOs, and governments (Edelman, 2024). Although trust declined by only 4% overall (Newman et al., 2024), this reflects a broader, ongoing trend that intensifies during political crises and conflicts (Johnson, 1993). For example, trust in media fell 13% in Spain and Greece during the EU crisis (Köhler and Otto, 2018) and 20% in Egypt after the Arab Spring (Hanitzsch et al., 2018). Recent studies show the pandemic accelerated this decline, with more pronounced effects at individual and national levels (Adam et al., 2023). In Austria, the case study of this research, media trust dropped from 45.1% in 2017 to a record low of 34.9% in 2024 (DNS Network Austria, 2024).

Moreover, the industry suffered a global loss of US$63 billion in advertising revenue during the pandemic’s first year (WARC, 2020), highlighting the media’s excessive reliance on digital advertising and accelerating efforts towards subscription-based revenue models and paid content strategies to offset losses (Jenkins, 2020). While digital subscriptions surged initially, leading publishers to prioritize subscriptions over advertising by 2023 (Newman et al., 2023), this optimism waned due to widespread consumer financial constraints (Fletcher, 2020). The proportion of individuals willing to pay for online news stagnated at 17%, a trend continuing into 2024 for the third consecutive year (Newman et al., 2024). In Austria, this stagnation is even more pronounced, with the willingness to pay (WTP) for online news dropping by half a point to 13.7% in 2024, compared to the previous year (DNS Network Austria, 2024).

Accordingly, to restore the media industry’s socio-economic stability in the post-pandemic era, addressing media trust and WTP is essential. The pandemic has reshaped the media landscape, making it crucial to understand how trust and WTP interact to build a resilient sector. This paper explores this relationship and identifies potential predictors and moderators that influence it.

## The issue of „media trust“

The concept of “trust” holds significant relevance in various forms of social interaction, playing a central role across different relationship levels. Indeed, trust involves establishing a connection with individuals, groups, or institutions built on the expectation, albeit not guaranteed, that they will act in one’s best interest (Delhey and Newton, 2003). Therefore, it serves as the “foundation of the social relationship that we call citizenship” (Coleman, 2012: 36).

If media trust is understood as “the willingness of the audience to be vulnerable to news content based on the expectation that the media will perform in a satisfactory manner” (Hanitzsch et al., 2018: 5), it becomes clear that trust is essential not only for society’s general sense of ontological security and the stability of the media industry, but also for democratic citizenship, fostering an informed public capable of political engagement (Jakobsson and Stiernstedt, 2023). However, trust should not be unconditional; it must include a healthy dose of criticism, grounded in critical media literacy for objective analysis (Clark, 2013). In democracies, media trust sustains the public sphere, promotes citizen engagement, and acts as a watchdog (Habermas, 1962; Curran, 2005). Without it, media lose democratic legitimacy, weakening both the public sphere and democracy. Thus, while a certain level of skepticism is necessary, media can only fulfill their role if trusted (Vara-Miguel et al., 2023).

This alone would legitimize the (academic) interest in investigating media trust, which has a long research tradition (Fisher, 2016). However, the issue extends beyond the “hostile media effect” (Gunther et al., 2017), where readers disapprove media´s “implicit or explicit criticism of their heroes” (Schudson, 2022: 150). Indeed, trust involves uncertainty and a leap of faith, as it comes into play in situations where decisions cannot be fully calculated (Möllering, 2006). It involves an exchange of promises and expectations, along with the willingness to take risks based on uncertain positive outcomes (Flores and Solomon, 1997). This is particularly true for media, as audiences struggle to cross-verify content and assess media professionals’ intentions, complicating fairness evaluations (Tsfati and Cohen, 2012). The digital age’s overwhelming “tsunami” of information further impedes trust, as citizens face challenges in navigating the “info-smog” and ongoing issues like “fake news,” algorithm manipulation (Flew and Jiang, 2021), and the COVID-19 “infodemic” (Wardle, 2021). Yet, media itself has contributed to this erosion through poor reporting, hoaxes, plagiarism, and daily inaccuracies (Peters and Broersma, 2013).

Additionally, as traditional trust-based and experience goods, media lack transparency regarding quality. Consumers derive value from the consumption experience itself (Cooper-Martin, 1991), meaning they purchase media content without prior knowledge of its quality and potential benefit (Nelson, 1970). This inability to evaluate benefits beforehand undermines rational decision-making, making trust essential. As a result, media must overcome this challenge by encouraging a greater leap of faith from consumers and using additional mechanisms to persuade them to purchase content whose quality can only be assessed post-consumption.

Addressing media trust thus requires a holistic approach that considers both dimensions. First, it involves trust in media institutions and their integrity in fulfilling societal roles, including journalistic practices like topic selection, fact-checking, research, and ensuring accuracy and fairness (Blöbaum, 2014). This in turn involves understanding media trust as trust in media sources, channels, and content, which includes assessing formal qualities like completeness, conciseness, consistency, and objectivity, alongside evaluative attributes such as accuracy, authenticity, and credibility (Appelman and Sundar, 2016; Gaziano and McGrath, 1986). Second, it also requires considering the leap of faith consumers make when engaging with media content. This involves building trust in media brands, which helps consumers reduce uncertainty when choosing from competing options (Chaudhuri and Holbrook, 2001), ensuring the financial viability of media offerings (Fisher, 2016). Indeed, media brands – defined by recognizable characteristics that evoke certain associations (Kim et al., 2010) – offer a crucial recognition factor amid rising costs, competition, and consumer fragmentation. This recognition can translate into a competitive advantage (Chan-Olmsted, 2011; Chan-Olmsted and Kim, 2023) by influencing purchase intent, willingness to pay, and overall consumption (Chaudhuri and Holbrook, 2001; O’Brien, 2022; Schranz et al., 2018).

## The economic value of media trust

As many media companies struggle for financial viability, trust in media (brands) is crucial for competitiveness (Chan-Olmsted and Kim, 2023) and building sustainable revenue (Toff et al., 2020). Trust is therefore a valuable asset for enhancing reputation and achieving financial sustainability (Vanacker and Belmas, 2009). A trustworthy media landscape also benefits the advertising industry through the halo effect (Liu-Thompkins, 2019), improving advertising effectiveness and helping ad-financed media achieve better financial results (Chan-Olmsted, 2011).

As previously discussed, trust is inherent in all social interactions that embody value (Delgado-Ballester and Munuera-Alemán, 2005) and plays a key role in business exchange relationships. In a fragmented and unpredictable media landscape, trust can mitigate consumer uncertainty and create a competitive advantage, influencing consumption, decision-making, purchase intent, and preference for products within the same category, encouraging higher payments for trusted products (Chaudhuri and Holbrook, 2001). Indeed, previous studies show consumers are more willing to pay for news from trusted brands (Myllylahti and Treadwell, 2022). Therefore, from an economic perspective, focusing on trust is valuable, as willingness to pay reflects consumer preferences, which determine a good’s value. In other words, the higher the willingness to pay, the greater the perceived value of the good (Breidert, 2006).

This assumption is based on microeconomic consumption and preference theories. Consumption theory explains how demand for goods is influenced by external determinants like the prices of goods and consumer income, which dictate whether a consumer can afford a desired bundle of goods and how much they will actually purchase. It also considers subjective factors, which, in economic terms, are reflected in consumer preferences (Deaton and Muellbauer, 1999). This is where preference theory comes into play, examining how people choose from different alternatives to maximize satisfaction or utility (Samuelson, 1948), considering preference ordering, the rationality assumption, and budget constraints. Simply put, under the rationality assumption, consumers select the bundle of goods that provides the highest utility within their budget (Deaton and Muellbauer, 1999; Hausman, 2012).

In the post-pandemic era, where economic constraints and rising inflation have limited consumer resources (Fletcher, 2020), understanding how consumers prioritize spending is crucial. Under the rationality assumption, this poses a dual challenge for media: the aforementioned lack of transparency in quality and the availability of free news on social media platforms. Indeed, platforms can engage readers by providing (journalistic) content for free, as they bear no production costs for it. Simultaneously, they attract advertisers with the opportunity to target a vast number of potential consumers in a specific and personalized manner. Consequently, this has cultivated a “freebie” mentality among users/consumers who now expect and, to some extent, demand unrestricted access to media (journalistic) content on the Internet (Goyanes et al., 2020). This shift undermines the advertising-financed revenue model that once supported the press, accelerated by digitalization and the pandemic (Dyomkin, 2021). But: despite news organizations pivoting to paid subscriptions to offset lost advertising revenue (Jenkins, 2020), consumers’ willingness to pay for online news remains low.

From the perspective of rational economic behavior, given budget constraints, consumers are unlikely to pay for content when free alternatives exist. Therefore, media companies must not only explore alternative ways to monetize online content but also effectively convey its value to persuade consumers to pay a non-zero price. This necessitates addressing complex issues around quality, perceived value, and trust (Flew, 2021). Thus, this study aims to explore the connection between media trust and willingness to pay, while also identifying potential factors that influence this relationship, by addressing the following research question(s):

RQ: *How does trust in the media influence the willingness to pay for online news content? What are predictors and what role do potential moderators play in this relationship?*

## Previous research

As evident from the theoretical framework, a considerable body of research addresses questions surrounding trust in media and willingness to pay (WTP). However, there is a notable lack of studies explicitly examining the correlation between these two factors (Vara-Miguel et al., 2023). O’Brien et al. (2020) highlight this gap, noting that no quantitative study specifically explores the relationship between media trust and past payment (PP), payment intent (PI), or WTP for digital journalism. This gap persists despite research on trust in e-commerce suggesting a strong influence on payment intent, and the recognition that many highly successful digital news brands are perceived as trustworthy. These observations could be interpreted “as evidence of a positive relationship between trust and WTP/PI” (O’Brien et al., 2020: 666). Supporting this perspective, Schranz et al. (2018) found that higher trust in the media system correlated with a greater WTP for news. Additionally, existing research confirms that media skepticism is negatively correlated with payment behavior (Goyanes et al., 2023; Lee et al., 2015), while media trust emerges as a key predictor of WTP (Park et al., 2022; Yu et al., 2021).

Accordingly, we hypothesize:

H1: There is a significant positive correlation between trust in the media and willingness to pay.

Research on media trust and WTP has thus far taken various approaches. However, the overarching aim and research interest lie in understanding the factors that influence trust in the media and the WTP for online journalistic content.

In this regard, Tsfati and Ariely’s (2014) study on media trust, based on the World Value Survey across 44 countries, identified key predictors of trust at various levels of abstraction. They emphasized the significance of audience trust in the news media, noting its influence on media selection and audience response, while also highlighting the lack of correlational studies on media trust. At the micro level, they examined the effects of socio-demographic factors and found that women tend to trust the media significantly more than men, supporting findings from previous studies such as Jones (2004), Schranz et al. (2018), Kalogeropoulos et al. (2019), and Andersen et al. (2023). However, other studies, including Gronke and Cook (2007) and Livio and Cohen (2016), reported the opposite, suggesting men trust the media more, while Bennett et al. (2001) found no significant gender effect on media trust.

In their systematic literature review, O’Brien et al. (2020) explore consumer-based factors influencing PP, PI, and WTP for online news, noting varying and sometimes contradictory results. For instance, some studies find that being male positively affects PP (Casero-Ripollés, 2012; Punj, 2015), PI (Chyi, 2012; Chyi and Lee, 2013), and WTP (Goyanes et al., 2023), with Flew (2021) suggesting that middle-aged men are most likely to subscribe to and pay for news. However, other studies show that women may have a higher WTP (Goyanes, 2015), while some argue that the variable “gender” has no significant impact on WTP overall (Goyanes, 2014; Ye et al., 2004). Nevertheless, despite the lack of consensus, it can be assumed that “gender” does have some influence on general WTP (O’Brien et al., 2020).

Previous studies also show inconsistencies regarding other socio-demographic factors. While the coefficient for age was deemed insignificant in Tsfati and Ariely’s (2014) investigation, Andersen et al. (2023) observed a statistically significant correlation between the variable “age” and the linear trajectory of alternative news orientation. Schranz et al. (2018) contradicted this, noting that although younger people tend to mistrust the media more, age does not have a linear effect on trust. Furthermore, Kalogeropoulos et al. (2019) found that while older individuals showed a stronger link between legacy media use and trust, age did not moderate the relationship between digital the use of digital websites and trust in news.

The variable “age” also lacks a clear pattern regarding general WTP for online news. For instance, Goyanes (2014, 2023) found younger people more willing to pay. However, this contradicts his 2015 study on WTP for local journalism (Goyanes, 2015). Similarly, Chyi (2012), Chyi and Lee (2013), and Fletcher and Nielsen (2017) suggest payment intent decreases with age, while Ye et al. (2004) found the opposite. Other studies (Beier et al., 2018; Punj, 2015) consider the variable “age” statistically insignificant. O’Brien et al. (2020) conclude that age negatively influences PI for general content but call for further research to clarify its impact on WTP and PP.

Another socio-demographic variable often examined is “education”, which tends to exhibit a negative correlation with trust in the media (Kalogeropoulos et al., 2019; Tsfati and Ariely, 2014), but a positive correlation with general WTP (Punj, 2015). However, differentiated analysis concerning PP, PI, and WTP remains largely underexplored (O’Brien et al., 2020).

Despite the inconsistencies, it is likely that socio-demographic factors influence both media trust and WTP. Thus, we propose the following hypothesis:

H2a: Socio-demographic factors predict trust in the media and the willingness to pay.

H2b: Socio-demographic factors moderate the correlation between trust in the media and willingness to pay.

At the micro-level, media use is also often analyzed in relation to media trust and WTP. Some studies view trust as a predictor of media exposure (Tsfati and Cappella, 2003), while others see exposure as a predictor of trust (Jackob, 2010). However, the direction of influence between media trust and media use remains unclear (Strömbäck et al., 2020).

Nevertheless, there is general agreement that regular news media consumers tend to trust the media more than non-consumers (Kiousis, 2001). This is partly due to the reinforcing effect of repeated exposure: the more frequently individuals are exposed to and engage with news media, the greater their tendency to trust these sources (Tsfati and Ariely, 2014). Additionally, this phenomenon is linked to the concept of familiarity which often correlates with trust (Misztal, 1996). Essentially, the more familiar we are with something or someone, the more likely we are to develop trust in them (Jakobsson and Stiernstedt, 2023).

While recognizing that media trust interacts with other needs during media content selection and some people may choose to consume media despite their skepticism due to a strong need for cognition (Tsfati and Cappella, 2005), from a rational audience perspective, it appears illogical for individuals to engage with media they do not trust (Tsfati and Cappella, 2003). Previous research increasingly suggests a positive link between media trust and greater use of legacy media (Jackob, 2010; Kiousis, 2001; Tsfati and Cappella, 2003), as well as between interest-driven consumption and trust (Park et al., 2022; Schranz et al., 2018; Vara-Miguel et al., 2023). Therefore, trust and exposure are expected to correlate (Strömbäck et al., 2020), though some studies report a modest or insignificant relationship (Bennett et al., 2001; Ladd, 2012).

Regarding WTP, more intensive media use seems to positively influence it (Goyanes, 2014; 2015; Fletcher and Nielsen, 2017; Fletcher, 2020; O’Brien et al., 2020; Park et al., 2022; Goyanes et al., 2023). Thus, we propose the following hypothesis:

H3a: Media use predicts trust in the media and the willingness to pay.

H3b: Media use moderates the correlation between trust in the media and willingness to pay.

However, especially trust in the media is not only associated with factors at the micro-level, such as demographics or media use. Various studies have explored macro-level factors and underscored the close relationship between trust in politics and government and trust in the media. Consequently, individuals who trust democratic institutions are more likely to trust the media, and vice versa (Andersen et al., 2023; Lee, 2010). This is because sitizens rely on the media for accurate information to make informed political decisions (Coleman, 2012). Without trust in political information, confidence in democratic processes erodes (Tsfati and Cohen, 2005). Indeed, previous studies have also demonstrated a strong correlation between media trust and trust in democracy (Tsfati and Ariely, 2014), and approval of political institutions (Hanitzsch et al., 2018). Furthermore, political partisanship is also positively associated with WTP for online news (Lee et al., 2022). Thus, we propose the following hypothesis:

H4a: Trust in policy predicts trust in the media and the willingness to pay.

H4b: Trust in policy moderates the correlation between trust in the media and willingness to pay.

## Method

To test the hypotheses and research question, the Austrian Gallup Institute conducted Computer Assisted Web Interviews through their online panel (gallupforum) in Austria from October 5 to 18, 2023. The gallupforum operates in compliance with the international standard for market, opinion, and social research ISO20252^1^. Online panel surveys have gained popularity in the past decade due to their speed, cost-effectiveness, elimination of interviewer bias, and the flexibility they offer respondents, reducing intrusiveness and the social desirability effect (Fisher, 2005).

The final weighted sample, after a pretest, included *n = 1000* individuals aged 16+, representing Austria’s web-active population, balanced by gender (49% male, 51% female^2^), age, income, and education. The survey covered media usage, trust, perceptions of media performance, payment behaviors, willingness to pay, and the relationship between media and politics, along with trust in political institutions.

To test the hypotheses, multiple regression analyses with listwise deletion were conducted. Predictor selection followed theoretical considerations, and interaction terms were included to examine moderation effects between media trust and gender, trust in politics and media use. Non-standardized coefficients, standard errors, and adjusted R^2^ values (for model fit) were reported. Predictors were considered significant if *p* < .05. All analyses were performed in Stata 16.

### Measures

*Media trust* (MT) was measured through source credibility (SC) and message credibility (MC) as proposed by Fisher (2016). This approach assumes that a medium is trusted when its information consistently proves credible (Van Dalen, 2020). Credibility, in this context, refers to the perceived believability of information or its source (Bentele and Seidenglanz, 2008). While credibility focuses on the perceived truthfulness of information and its source, trust is a broader concept, involving expectations about media fulfilling societal roles.

Encompassing the expectation that media will fulfill societal roles (Van Dalen, 2020). Therefore, in this study, credibility – divided into MC and SC – is used as a measurable precursor to trust. In line with our theoretical framework, MC relates to confidence in content quality, while SC reflects trust in the media brand. SC was measured by asking respondents to rate (1 = not at all credible, 5 = very credible) the credibility of news reporting from different media brands (see Appendix A), following the methodologies from the Pew Research Center, Reuters Digital News Report, and Edelman Trust Barometer surveys (Edelman, 2024; Fisher, 2016) used in prior research (e.g., Adam et al., 2023). MC was assessed by agreement with statements on reporting credibility, fairness, balance, public guidance, and overall satisfaction (*α = 0.907*; see Appendix A), based on factors identified by Gaziano and McGrath (1986) and Meyer’s (1988) credibility index. Finally, MT was calculated by normalizing SC and MC to a 0-1 scale and averaging these two variables.

*Willingness to pay* (WTP) was assessed by asking respondents if they had ever paid (PP) or would consider paying (WTP) for digital subscriptions, individual articles, and specific content. The composite scale showed acceptable internal consistency (*α = 0.756*), with higher scores indicating greater WTP. Furthermore, participants also reported their current monthly expenditures (media expenses (ME)) on media products to investigate whether factors affecting WTP also manifest in actual spending behavior or solely impact intention.

*Media use* (MU) was measured by asking participants how much time they had spent the previous day informing themselves about current events. Based on the distribution of responses, the time spent was then divided into two categories: up to 1 hour and 1 hour or more.

*Political trust* (PT) was measured by asking respondents to rate their level of trust in political institutions, political parties, and politicians on a scale from 1 (very low) to 5 (very high). The composite scale exhibited high internal consistency (*α = 0.936*).

As *socio-demographic factors* to be examined in the model, we included the following variables: gender^3^, age, educational level (ranging from no graduation/compulsory to university), monthly net (equivalized) household income, and the region of residence (differentiating between countryside and cityside).

## Results

### Descriptive statistics

When participants were asked to rate SC, the combined mean score across all media brands was 3.70, with public service media regarded as highly trustworthy (ORF 58%, ZDF 28%). Among media types, television had the highest credibility (*M = 3.98*), followed by radio (*M = 3.95*) and newspapers (*M = 3.79*). Social media was rated least credible (M = 2.91).

For MC, 53% of respondents found media reporting credible, 50% were satisfied with it, and 45% considered it a reliable guide. However, WTP was limited: 13% had subscribed to a digital medium, and 15% considered it. WTP for individual articles was 18%, with only 5% having paid. Monthly expenditure (ME) was highest for printed daily newspapers (€28), while its online offerings received €16.

Media usage is widespread, with 80% of respondents using media multiple times daily. This increases to 90% in wealthier households and decreases to 73% in less affluent ones. Even among those who felt poorly informed about current events, 75% still used media several times a day.

Television is the most frequently used medium for news (74%), followed by newspapers (58%) and radio (56%). Social media is also significant, with 52% using it for news. Despite high media usage, about 25% of respondents often avoid news, especially younger ones (39% of ages 16-30). Psychological stress (36%) and lack of trust in the media (18%) were the primary reasons for news avoidance.

Trust in political institutions is low, with only 12% expressing (very) high trust. The majority (51%) reported low to no trust, while 37% had moderate trust.

### Correlations

**Figure 1.**
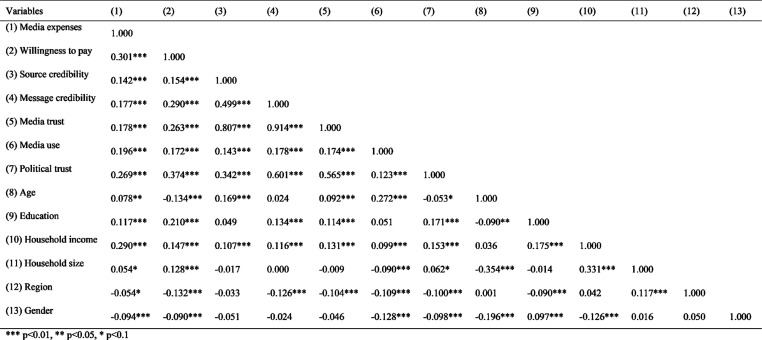
Pairwise correlations.

Figure 1 presents the pairwise correlations among the different variables and it shows a significant correlation between media trust and WTP (*r* (885) = 0.263, *p* < .001), as well as between media trust and media expenses (ME) (*r* (901) = 0.178, *p* < .001), thus supporting **H1**. Additionally, WTP correlates with ME (*r* (940) = 0.301, *p* < .001), suggesting that WTP moderately translates into actual spendings on media.

Interesting are the correlations involving *political trust*. It shows a moderate correlation with both WTP (*r* (959) = 0.374, *p* < .001) and ME (*r* (979) = 0.269, *p* < .001), and a strong correlation with media trust (*r* (919) = 0.565, *p* < .001), which becomes even stronger when considering the single variable of message credibility (*r* (975) = 0.601, *p* < .001). Media use has weak but significant correlations with WTP (*r* (937) = 0.172, *p* < .001) and ME (*r* (956) = 0.196, *p* < .001), suggesting a slight increase in WTP with increasing media use, though causality cannot be inferred. This pattern also applies to the socio-demographic factors, which show statistically significant but weak correlations, some of which are negative, with both WTP and ME. Consequently, further analyses were conducted to gain a better understanding of the factors influencing WTP and ME.

### Regression analyses

**Figure 2.**
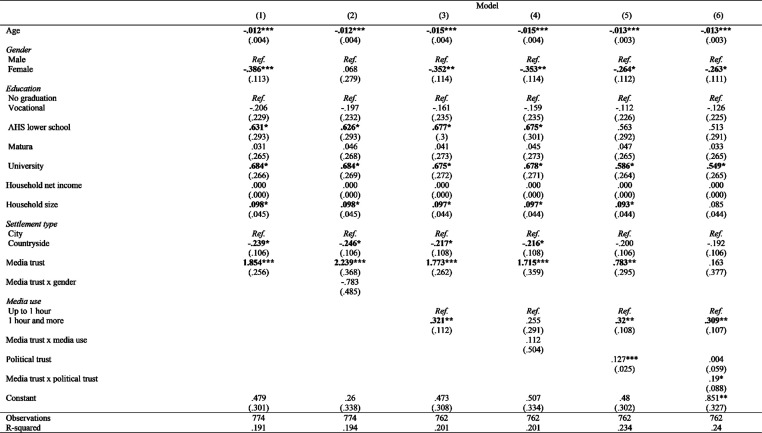
Predictors of WTP (with media trust as IV).

The regression analysis in Figure 2 shows regression models (1 to 6), with WTP for media content as the dependent variable, and media trust as the primary independent variable. Each model includes different predictors to assess their impact on WTP. The models explain 19.1% to 24.0% of the variation in the dependent variable (R^2^_adj_ * 100), indicating an acceptable fit (Ozili, 2022).

In Model 1, we examine the effects of socio-demographic factors on WTP. The results show that higher education and larger household sizes are linked to higher WTP, while older age, being female, and living in rural areas are associated with lower WTP. These patterns persist even after controlling for household income and size, indicating that they operate independently of equivalent income. This supports **H2a**, suggesting that socio-demographic factors are direct predictors of WTP. However, in Model 2, adding an interaction term between media trust and gender reveals no significant effect, meaning gender does not moderate the relationship between media trust and WTP. Thus, **H2b** is not supported.

In Model 3, media use is introduced as a predictor. The results show that individuals who use media more intensively (i.e., for 1 hour or more) have significantly higher WTP compared to those who use it for less than an hour (B = .321, *p* < .01). This effect remains significant across several models, supporting **H3a**. However, the interaction term between media trust and media use in Model 4 is not significant, indicating that media use does not moderate the media trust-WTP relationship, thus rejecting **H3b**.

In Model 5, political trust is added and emerges as a significant predictor of WTP. This supports **H4a**, as political trust independently predicts WTP even after accounting for socio-demographic variables.

Finally, in Model 6, the interaction between media trust and political trust is included to test if political trust moderates the media trust-WTP relationship. The positive and significant coefficient (B = .19, *p* < .05) indicates that higher political trust enhances the positive effect of media trust on WTP, supporting **H4b**.

Across all models in Figure 2, media trust consistently predicts WTP, further supporting **H1**. The strongest relationship is seen in Models 1 and 2, where socio-demographic factors are the main controls. However, as additional moderators like political trust are introduced in Models 5 and 6, the effect of media trust on WTP weakens slightly, indicating that the relationship is nuanced and may be influenced by other socio-political factors.

**Figure 3.**
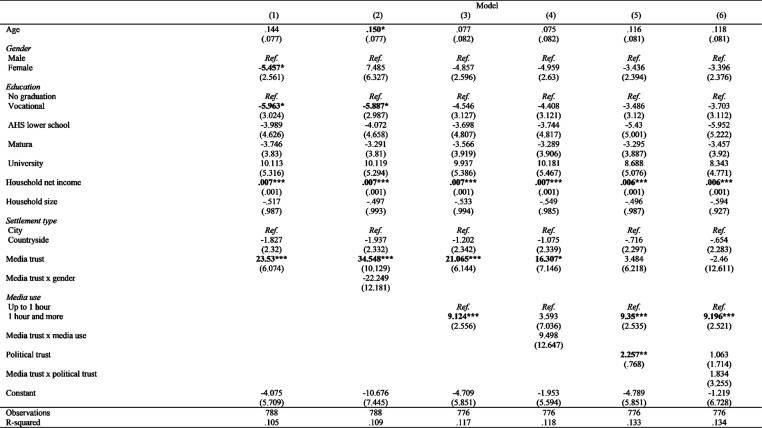
Predictors of media expenses (with media trust as IV).

Using the same regression models, but with media expenditure (ME) as the dependent variable instead of WTP, a similar pattern of results emerges, though with lower explanatory power (10.5% to 13.4% variance explained), as some effects significant for WTP fail to reach statistical significance for ME (see Figure 3). In Model 1, gender is a significant predictor, with women spending less on media than men (B = −5.457, *p* < .05). However, this effect loses significance in the later models, and the interaction term included in Model 2 does not support the moderation hypothesis, as it is not statistically significant.

As expected, income positively and significantly influences ME, while other socio-demographic factors do not reach statistical significance. Media use and political trust (introduced in Model 3 and 5) remain significant predictors of ME, indicating that intensive media use and political trust are linked to greater spending. However, the interaction terms (media trust X media use in Model 4 and media trust X political trust in Model 6) do not reach significance, failing to support the moderation hypotheses.

Notably, media trust remains a significant predictor for ME in the simpler models (1 to 4) but loses significance in the more complex models (5 and 6) when political trust is included.

## Discussion

This study explored the relationship between media trust and WTP, focusing on various predictors and moderators. It was based on the premise that media trust is essential from both societal and economic perspectives. In democracies, media fulfill essential public functions, requiring consumer trust for credible information and guidance, especially in times of and post-crisis. Furthermore, trust is also economically essential, as its absence can cause financial instability due to a lack of transparency in media quality. Our findings strongly support this, with both correlation analyses and regression models showing that media trust significantly influences WTP and ME, emphasizing its key role in shaping consumer payment behavior.

The strong link between media trust, WTP, and ME highlights its relevance in consumer decision-making, particularly in the post-pandemic context, where the need for trustworthy media has grown due to rising skepticism driven by misinformation. This context has likely amplified the influence of media trust as a significant predictor of WTP. Indeed, during COVID-19 media consumption and trust patterns changed. The information overload, often contradictory or false, has led to confusion and a perception of media as biased, politically or commercially motivated, especially on sensitive topics like COVID-19 (Vara-Miguel et al., 2023). In addition, the rise of social media as an information source has also weakened traditional media’s role as intermediaries. In the post-pandemic scenario, the media industry must not only restore trust and encourage payment for content, but also meet the expectations for high-quality and reliable digital information.

Our findings indicate that consumers prioritize credible media (brands), which strongly influences both their intention to pay (WTP) and their actual spending behavior (ME). Consequently, especially legacy media, must focus on (re-)building trust to secure financial support from consumers, despite the availability of free journalistic content on social media. Achieving this aim requires a comprehensive strategy that addresses both aspects of media trust: reinforcing the unique value and quality of media content, while also strengthening trust in media brands to guide consumer preferences toward paid content over free alternatives.

In order to achieve the former, existing literature highlights transparency as a key strategy (Garusi and Leonhardt, 2024; Toff et al., 2020; Uth, 2024; Zahay et al., 2021). Transparency, defined as providing an unbiased view of behind-the-scenes processes, can help regain trust from skeptical audiences (Uth, 2024). This requires a holistic approach within media organizations, ensuring both the visibility and inferability of content (Michener and Bersch, 2013). This requires making information complete and easily accessible, allowing users to verify its accuracy. Technologies like blockchain can support this by guaranteeing transparency in sourcing and content integrity.

Accountability is also crucial for building media trust, requiring strict ethical standards in sourcing, verification, and conflict-of-interest management. Media outlets can boost credibility by addressing inaccuracies swiftly and appointing independent ombudsmen. Thus, to enhance trust, media organizations must uphold quality standards and follow traditional journalistic principles such as objectivity, accuracy, balance, and diversity (Garusi and Leonhardt, 2024; Zahay et al., 2021). Fact-checking, clear labeling, and prioritizing in-depth reporting over sensationalism also help restore trust.

Lastly, a key strategy for restoring and building trust, as highlighted in the literature, is audience engagement (Zahay et al., 2021). Actively involving audiences in journalistic processes and valuing their perspectives aligns media content with expectations, fostering greater satisfaction, loyalty, and trust (Koliska et al., 2023; Uth, 2024).

Beyond the critical importance of media trust for WTP and ME, it is essential to understand the factors influencing financial support from the audience. Our findings offer nuanced insights, aligning with and diverging from previous research on socio-demographic factors, while stressing the need for tailored strategies for different audience segments. Here, implementing flexible and inclusive pricing strategies, such as micropayments, can increase WTP and reach diverse socio-demographic groups. Furthermore, our findings confirm that regular news consumers tend to trust media more and are more inclined to pay for it. Utilizing advanced data analytics, AI, and machine learning can thus facilitate tailored content recommendations and customized newsfeeds, thereby boosting engagement, loyalty, and WTP.

While enhancing the quality and relevance of media content is crucial for (re-)building trust, we believe this approach may fall short from a media economics perspective. It is equally important to shape consumer preferences to increase WTP and ME. After all, low WTP may not always reflect poor journalism, but rather a failure by media companies to build strong brands and engage in effective branding and marketing activities.

Indeed, while product quality influences the price consumers are willing to pay, the strength of a brand is crucial for its competitive success. Strong brand credibility influences consumer brand choice, driving higher prices and perceived product uniqueness (Dwivedi et al., 2018). According to choice theory, perceived uniqueness simplifies decision-making by distinguishing a brand from competitors, adding value and increasing willingness to pay a premium price (Dhar and Sherman, 1996).

While marketing and psychology research (e.g., Sung and Kim, 2010) highlight the importance of strong brands and brand trust in shaping consumer preferences, this perspective remains understudied in media management. Despite Chan-Olmsted’s (2011) call for media companies to adapt their marketing strategies to survive in evolving market conditions, research in this area is still limited. Therefore, we advocate for enhancing the internal quality of journalism alongside a focus on implementing effective marketing strategies to cultivate strong media brands.

## Limitations and further research

Despite its valuable contribution, the study has limitations. Media trust varies significantly across countries, requiring country-specific analysis (Hanitzsch et al., 2018; Newman et al., 2023, 2024). While this justifies our focus on Austria, it limits generalizability to other regions and demographics. Consequently, some results may hold more explanatory value within Austria’s specific political and social post-pandemic contexts. To address this, we plan to conduct longitudinal studies to examine how these relationships evolve over time, allowing broader generalizations.

The variables in this study were selected based on their relevance in existing literature and their potential to moderate the trust-WTP relationship. While this study took an exploratory approach, future research should include additional factors that may influence consumer behavior. Moreover, while the homo economicus model offers valuable insights into consumer behavior, it may oversimplify the complexities between media trust and consumption/WTP. Future research should address this gap to provide a more comprehensive understanding of the underlying mechanisms.

Furthermore, while our study found a strong correlation between political trust and media trust (see Figure 1), media trust loses significance when political trust is included, suggesting they capture similar aspects of respondents’ attitudes. Future research should explore this relationship further to provide a clearer understanding. Additionally, given the diminished credibility scores observed for social media in our study, there is a need to explore how these platforms influence trust and credibility in the broader media landscape.

Lastly, we emphasize the need for future studies to explore the duality of “media trust”, considering both content quality (MC) and media brand roles (SC). Our study’s single-scale measurement is a limitation, so future research should build on the dimensions developed by Chan-Olmsted and Kim (2023) and Heim et al. (2023). This will offer a more nuanced understanding of consumer perceptions of media brands and their impact on trust, willingness to pay, and media spending.

## Supplemental Material

Supplemental Material - Trust has a price?! Unraveling the dynamics between trust in the media and the willingness to pay in the post-pandemic scenario Supplemental Material for Trust has a price?! Unraveling the dynamics between trust in the media and the willingness to pay in the post-pandemic scenario by Denise Voci, Matthias Karmasin, Sonja Luef, Sandra Förster and Andy Kaltenbrunner in Journalism

## Notes

1 For further details on panel management, quality assurance, recruitment methods, and data security, please refer to https://www.gallup.at/de/online-panel/gallupforum/.

2 It has to be noted here that respondents were provided with the option to self-identify as “male”, “female” or “diverse”. However, only a single respondent opted for the “diverse” category.

3 See footnote No.2.
