# Supplementary material for: Trust has a price?! Unraveling the dynamics between trust in the media and the willingness to pay in the post-pandemic scenario
Source: Journalism (Lond). 2024 Dec 23;27(3):563–84. doi: 10.1177/14648849241311101 (PMC12890083; doi:10.1177/14648849241311101)
Supplement: Supplemental Material - Trust has a price?! Unraveling the dynamics between trust in the media and the willingness to pay in the post-pandemic scenario [file sj-pdf-1-jou-10.1177_14648849241311101.pdf]

Appendix A – Variables and measurements

Variable Name	Questionnaire question (s)	Items	Type	Range
Gender	First, a few questions about yourself:<br>What is your gender?	1 = Male<br>2 = Female<br>3 = Diverse	Categorical	-
Age	What is your exact age?	1 = Exact age: ____<br>2 = Prefer not to answer	Continuous	17 - 86
Education	What is the highest level of education you have completed?	1 = No formal qualification, compulsory education<br>2 = Vocational/technical/middle school<br>3 = AHS lower secondary school/secondary school without Matura<br>4 = Matura<br>5 = University/university of applied sciences	Categorical	-
Household net income	What is your household's net monthly income?	1 = Under €1.000<br>2 = €1.000 - €1.500<br>3 = €1.501 - €2.000<br>4 = €2.001 - €2.500<br>5 = €2.501 - €3.000<br>6 = €3.001 - €3.500<br>7 = €3.501 - €4.000<br>8 = Over €4.000<br>9 = Prefer not to answer	Continuous	750 – 4.250
Household size	How many people, including yourself, live in your household?	1 = 1 person (I live alone)<br>2 = 2 people<br>3 = 3 people<br>4 = 4 people<br>5 = 5 people<br>6 = More than 5 people	Continuous	1 - 6
Settlement type	Do you live in a...	1 = City<br>2 = Countryside	Categorical	-
Media expenses (ME)	How much do you currently spend on media consumption per month (streaming services, newspaper and magazine subscriptions, digital	Exact amount: ______	Continuous	0 – 610

	subscriptions, etc.)?<br>(Please enter an exact amount)			
Willingness to pay (WTP)	More and more online media have a “paywall” for their content, i.e. you have to pay for certain content or sign up a digital subscription. What have you already done or would consider in the future?<br>(Response options: Already done, Would consider, Would not consider)	1 = Sign up for a digital subscription<br>2 = Pay for individual articles<br>3 = Donate for selected content	Continuous	0 – 6 (higher values = higher WTP)
Source credibility (SC)	How credible do you consider the news about current events from the following media/sources?<br>(1 = Not credible, 5 = Very credible)	List of 53 different media outlets *(ORF, ZDF, RTL, VOX, ATV, PULS4, PULS24, PRO7, SAT1, ServusTV, ARD, Kabel 1, 3sat, BBC, oe24.TV, n-tv, Der Standard, Die Presse, Kronen Zeitung, Kurier, Heute, Österreich, Wiener Zeitung, Kleine Zeitung, OÖN, Salzburger Nachrichten, TT, VN-Vbg. Nachrichten, NÖN, BVZ, Mein Bezirk, Falter, Die ganze Woche, News, Profil, Trend, Gesünder Leben, Gewinn, Medizin Populär, Hitradio Ö3, Ö1, Krone Hit, FM4, Radio Wien, Antenne, Ö2, Radio Arabella, Life Radio, Facebook, Twitter (X), Instagram, YouTube, TikTok)*	Continuous	1 – 5 (higher values = higher SC)
Message credibility (MC)	To what extent do you agree with the following statements?<br>(1 = Strongly disagree, 4 = Strongly agree)	1 = The reporting about COVID-19 in the Austrian media was credible<br>2 = Overall, I am satisfied with the coverage about COVID-19 in the Austrian media<br>3 = The reporting about COVID-19 in the Austrian media was relevant to my life and provided guidance<br>4 = The reporting about COVID-19 in the Austrian media was fair and balanced	Continuous	1 – 4 (higher values = higher MC)
Media trust	Derived variable = Combination of Source Credibility (SC) and Message Credibility (MC)	Derived from SC and MC	Continuous	0 – 1 (higher values = higher trust)
Media use	How much time did you spend yesterday informing yourself about current news?	1 = No time at all<br>2 = Up to 1 hour<br>3 = Approximately 1 hour<br>4 = Approximately 2 hours<br>5 = Approximately 3 hours<br>6 = Approximately 4 hours	Categorical	-

		7 = Approximately 5 hours<br>8 = More than 5 hours		
Political trust (PT)	How much trust do you currently have in political institutions?	1 = Very low<br>2 = Low<br>3 = Medium<br>4 = High<br>5 = Very high	continuous	0 – 12 (higher values = higher PT)
	How much trust do you currently have in political parties?			
	How much trust do you currently have in politicians?			
